# Validation of the Chinese version of the scientific imagination inventory in primary school students

**DOI:** 10.3389/fpsyg.2026.1757878

**Published:** 2026-02-03

**Authors:** Duhong Peng, Junying Feng, Mingfeng Wang

**Affiliations:** 1 School of Education, Suzhou University of Science and Technology, Suzhou, China; 2 Suzhou High-tech District Experimental Elementary School, Suzhou, China

**Keywords:** Chinese primary school students, reliability, scientific imagination, validating, validity

## Abstract

Intensifying global competition in high technology and generative artificial intelligence (Gen AI) urgently calls for support to foster scientific imagination, which is essential for achieving breakthroughs in original innovation. As children enter primary school, their scientific imagination undergoes a rapid surge in development; however, few suitable tools are available to track and assess this growth. The Scientific Imagination Inventory (SII) demonstrated acceptable psychometric properties in samples of Korean students; however, its validity in other cultural contexts has remained unexplored. The present study was designed to evaluate the psychometric properties of the Chinese version of the SII, thereby addressing a critical gap in the availability of developmentally appropriate assessment instruments for primary school students in China. A total of 837 students in grades 2 to 6 were recruited from three private and public primary schools in Suzhou, China. Item analysis, exploratory factor analysis (EFA), confirmatory factor analysis (CFA), and criterion validity analysis were conducted. The results showed significant correlations between items and their respective dimension scores. EFA identified six factors that explained a cumulative variance of 55.99%. CFA supported a three-dimensional, six-factor structure (χ^2^/df = 1.553, RMSEA = 0.049, CFI = 0.906). Criterion validity was established through a significant correlation with the Vividness of Visual Imagery Questionnaire (VVIQ). Both internal consistency (*α* = 0.86) and split-half reliability (0.82) were satisfactory. The Chinese version of the SII showed adequate reliability and validity and is suitable for assessing the development of scientific imagination among primary school students in the sampled context. However, tests of measurement invariance indicated a lack of scalar invariance across gender and grade levels. Therefore, although the SII’s factor structure is generalizable to the Chinese context, our results underscore the critical importance of considering cultural and developmental response patterns when interpreting scores. Caution is advised against making direct mean comparisons across demographic groups.

## 1 Introduction

Scientists engage in imagination when thinking scientifically, such as Albert Einstein, Stephen Hawking, and Barbara McClintock (Fleer, 2023). They imagine constantly when generating research problems, designing experiments, interpreting data, troubleshooting, and drafting papers and presentations. Imagination is a crucial ability for scientists (Fleer, 2023). Nearly all major scientific discoveries are achieved through the verification of imaginative ideas (Zhang, 2023). Scientific imagination is the ability to construct images in the brain, generate ideas, and concretize these mental processes through the invention or creation of objects and products; this mental activity is not limited by rules or hindered by current modes of thought (Wang et al., 2014). Ho et al. (2013) proposed that scientific imagination emphasizes purposeful processes, defining it as the mental activity involved in creating new ideas that are consistent with scientific principles and are linked to daily life experiences. With generative artificial intelligence (GenAI) continuing to achieve breakthrough developments, the last possible advantage of human brain intelligence—imagination is being challenged. In this era, scientific imagination is becoming a pivotal skill. This ability is both crucial and fundamental in fostering elementary students’ inquiry skills; it enables them to conceptualize abstractions, conduct thought experiments, and devise creative solutions to challenges (Fleer, 2023; França et al., 2023).

Scientific imagination is a complex construct that integrates cognitive processes with epistemic and social assessments. Mun et al. (2013) characterized it in experienced scientists by identifying traits such as curiosity and a meticulous assessment of possibilities. Expanding upon this theoretical framework, Mun et al. (2015) subsequently operationalized the construct for a broader population, identifying and quantifying its three essential components—sensitivity, creativity, and productivity—in a large sample of Korean students. Analogical reasoning, mental simulations, and thought experiments are utilized by both experts and students, as noted by Clement (2008). The cognitive capacity for visualization underlies these abilities, especially mental simulation. Kozhevnikov et al. (2013) clarified this distinction by differentiating between object visualization and spatial visualization, the latter being fundamentally associated with scientific creativity.

Nevertheless, empirical research in this area has not advanced in alignment with theoretical progress. One potential reason is that existing studies have not yet developed a systematic, stable, and dedicated tool for assessing scientific imagination that is suitable for large-scale evaluation. Current research primarily follows two approaches: One derives insights from creativity theory to indirectly assess scientific imagination through divergent thinking tests or analyses of STEM project work (Guilford, 1967; Hu and Adey, 2002); the other relies on subject achievement tests and substitutes imagination assessment with the capacity to transfer scientific knowledge (Skorupiński, 2015). Both approaches overlook the developmental characteristics of “scientific imagination” as a distinct psychological construct and particularly lack specialized assessment tools for the cognitive traits of primary school students (Sawyer and Henriksen, 2024). The absence of appropriate tools directly contributes to challenges in identifying potential and diminishes the relevance of training, thereby exacerbating the structural misalignment between talent supply and demand. Consequently, a significant empirical gap remains: There is a lack of a systematic, large-scale assessment tool specifically designed to capture the unique developmental trajectory of scientific imagination in primary school students.

In this context, educators fail to accurately assess the real state of students’ scientific thinking, making it challenging to develop personalized training strategies for them (Shouse et al., 2007). Ultimately, this can negatively impact students’ interest in science and their spirit of inquiry, thereby limiting the development of their innovative capabilities (Gómez and Suárez, 2020; Kwangmuang et al., 2021; Lu et al., 2020). The development of scientific imagination is crucial in primary education, as this stage represents a critical period for the foundational establishment of students’ scientific interests and methodologies of scientific thinking (Koerber et al., 2015; Salahova, 2023; Sekaringtyas et al., 2024). If students’ scientific imagination is not effectively assessed and guided at this stage, its subsequent development will be restricted, making it difficult to establish a strong foundation for science learning in middle school or beyond (Osborne et al., 2003).

To address this gap and overcome the limitations of indirect measurement, the present study introduces and validates a Chinese adaptation of the Scientific Imagination Inventory (SII; Mun et al., 2015). The SII is a promising tool, as it is a dedicated, multi-dimensional instrument initially developed for student populations. By rigorously examining its psychometric properties within the Chinese cultural and educational context, this research aims to provide the field with a direct, standardized tool capable of assessing the core components of scientific imagination in elementary school students. Translating the SII into Chinese can generate distinct value in three key areas: (1) Developing the first scientific imagination assessment framework tailored to the Chinese cultural context, encompassing the entire continuum of “emotion-creation-reality,” thereby addressing the absence of standardized multi-dimensional tools for primary school students in China; (2) enabling direct application of the Chinese version of the SII in group assessments at the primary school level, enabling educational institutions and research organizations to obtain six sub-ability scores in approximately 15 min, thereby facilitating extensive early screening and longitudinal tracking; and (3) integrating the translated system into the existing national student physical health and science literacy monitoring platform after completing the translation, providing essential metrics for the educational evaluation framework. This study functions both as a cross-cultural adaptation of the assessment tool and as an investigation into early training pathways for cultivating innovative talent in the context of a future intelligent society.

## 2 Methods

### 2.1 Participants

Participants were 837 students recruited from three public and private elementary schools in Suzhou, China, using a combination of convenience and random sampling. This approach was chosen due to pragmatic constraints related to school access and administrative permissions, which are common in initial instrument validation studies. To partially mitigate sampling limitations and enhance the internal diversity of the sample, we deliberately selected schools of different types from various districts within Suzhou, aiming to capture a broad range of student backgrounds. A total of 850 questionnaires were distributed, yielding 837 valid responses after excluding invalid entries, corresponding to an effective response rate of 98.47%. The final sample comprised 837 participants, with 83 s graders (9.9%), 217 third graders (25.9%), 204 fourth graders (24.4%), 208 fifth graders (24.9%), and 125 sixth graders (14.9%). Of the total sample, 387 participants were female (46.2%) and 449 were male (53.6%). The entire sample was randomly divided into three subsamples.

Sample 1 comprised 414 participants randomly selected for exploratory factor analysis (EFA), including 34 third graders (8.2%), 123 fourth graders (29.7%), 132 fifth graders (31.9%), and 125 sixth graders (30.2%). Within this subsample, 193 were female (46.6%) and 221 were male (53.4%).

Sample 2 consisted of 321 participants randomly selected for confirmatory factor analysis (CFA), including 83 s graders (25.9%), 81 third graders (24.9%), 81 fourth graders (24.9%), and 76 fifth graders (23.7%). Among them, 148 were female (45.5%) and 172 were male (52.9%).

Sample 3 included 340 participants selected via convenience sampling from the overall dataset for reliability analysis, of which 328 questionnaires were valid.

### 2.2 Measures

#### 2.2.1 Scientific imagination inventory (SII)

The SII (Mun et al., 2015) is a 20-item self-report questionnaire. These items are organized into a three-dimensional, six-factor structure: (1) “scientific sensitivity” (SS), the driving force of imagination with two secondary factors of “emotional understanding” (EU) and “imagination experience” (IE), where the former emphasizes emotional resonance in the imagination process, while the latter highlights the transcendence of reality through interest and curiosity; (2) “scientific creativity” (SC), which focuses on problem discovery and solution strategies, including two secondary factors of “originality” (O) and “diversity” (D), which correspond to non-stereotyped novel thinking and multi-angle data exploration, respectively; (3) “scientific productivity” (SP), which focuses on the realistic transformation of imagination results, covering two secondary factors of “creative reproduction” (CR) and “scientific realism” (SR), which not only reflect the action tendency of transforming scientific knowledge into new solutions but also evaluate the rational judgment of the feasibility of imagination results. Each item is rated on a 5-point Likert scale, ranging from 1(totally disagree) to 5(totally agree). The score is computed as the mean of all factors, excluding organization. This average score ranges from 1 to 5, where elevated levels correspond to more pronounced perfectionist tendencies. For instance, in the Scientific Reality Sense dimension, Item 6 states: “I think events such as ‘Harry Potter’ can actually happen in.” A higher score on this item indicates that the participant has a clearer understanding that such imaginative events are unlikely to occur in the real world. Similarly, in the Imaginative Experience dimension, Item 14 (“I think about opposite situation to reality such as ‘if there is no air…’”) measures the tendency to engage in hypothetical thinking, with higher scores reflecting a greater ability to generate imaginative ideas based on personal curiosity, independent of real-world constraints. The instrument, developed by Mun et al. (2015), has demonstrated satisfactory internal consistency for both the overall scale and its subscales in adolescent populations.

#### 2.2.2 Vividness of visual imagery questionnaire

This measure assesses the ability to form and manipulate mental visual images in the absence of direct external visual stimuli. Visual imagination plays a significant role in scientific imagination, as scientists often use visualization to construct theoretical models, predict experimental outcomes, or comprehend complex scientific concepts (Dijkstra and Fleming, 2023). Visual imagination serves as a tool for simulating reality, enabling scientists to mentally “rehearse” scientific experiments or theoretical models (Shepard, 1988).

### 2.3 Procedure

To ensure equivalence between the Chinese and English versions of the SII, the *Guidelines for Cross-Cultural Adaptation* was followed (Beaton et al., 2000). The procedure included the following steps: First, forward translation was conducted. With authorization, two graduate students specializing in educational psychology and one faculty member in psychology, all with research experience and knowledge of measurement tool development, independently translated the original version, resulting in two versions, A1 and A2. The research team discussed and integrated these elements to create the preliminary translated version A. Subsequently, back-translation was performed. Two bilingual individuals, who had no prior exposure to the original scale, independently back-translated version A, producing versions B1 and B2. The research team compared and revised the back-translations to produce version B. Cultural adaptation was ultimately performed. Individuals possessing bicultural backgrounds engaged with the research team to conduct a comparative analysis of the original scale, version A, and version B. Modifications were made to enhance semantic clarity and align the items with local expression standards. Item 19 was initially translated as: “Reverse thinking is interesting, e.g., ‘If I were a girl (or boy)””. Following discussion, the example was revised to: “Reverse thinking is intriguing, for instance, ‘The day is dark, and the night is bright’.” The final Chinese version of the SII retained all original items and scoring methods.

### 2.4 Data analysis

Data analysis was conducted using SPSS 26.0 and Mplus 8.3. Item analysis, exploratory factor analysis (EFA), validity assessment, internal consistency checks, and test–retest reliability analysis were conducted using SPSS 26.0, whereas confirmatory factor analysis (CFA) was carried out in Mplus 8.3. Item performance was assessed using extreme-group comparisons and item-total correlation analysis. EFA was conducted using principal component analysis with varimax rotation. CFA was conducted using maximum likelihood (ML) estimation. Validity was assessed using Pearson correlation analysis. Internal consistency was evaluated using Cronbach’s *α* and split-half reliability coefficients. A *p*-value below 0.05 was deemed statistically significant.

## 3 Results

### 3.1 SII scores of the participants

The total SII score for the 837 primary school students was 3.55 ± 0.57. The score for the scientific sensitivity dimension was 3.68 ± 0.67, for the scientific creativity dimension was 3.61 ± 0.68, and for the scientific productivity dimension was 3.32 ± 0.68. Regarding item scores, Item 7 (“animals such as dogs and cats are able to feel emotions just like me;” M = 4.26, SD = 1.11) had the highest score, while Item 1 (“magic or wizardry is not real, but is trickery;” M = 2.28, SD = 1.28) had the lowest score.

### 3.2 Item analysis

The extreme-group test and the item-total correlation coefficient method were used to analyze the items of the second-order scale. First, the total score of each first-order dimension was calculated for the total sample, and the samples were sorted according to the score. The top 27% and bottom 27% of the samples were assigned to the high and low groups, respectively. Then, an independent samples *t*-test was performed between these two groups, and the results showed that there were significant differences between the high and low groups for all items (*p* < 0.0001). Next, correlation analysis was conducted between each item score and the total score of the corresponding first-order dimension. It was found that the critical ratio (CR) of all items was between 5.061 and 22.731 (*p* < 0.001), and the correlation coefficient between each item and its corresponding first-order dimension ranged from 0.22 to 0.64 (*p* < 0.01). Finally, correlation analysis between each item score and the total score of the corresponding second-order dimensions revealed that the critical ratio (CR) of all items was between 14.541 and 40.295 (*p* < 0.0001), and the correlation coefficient between each item and its corresponding second-order dimension ranged from 0.51 to 0.78 (*p* < 0.01). The item analysis indicated that the screening criteria required a CR value greater than 3 and an item–total correlation coefficient (r) exceeding 0.30 (Wu, 2010). Item 1 in the first-order model did not meet the criteria, but all items in the second-order dimensions met the criteria. Considering that Item 1 meets the item analysis standards for the second-order dimension and demonstrates sufficient discriminative power in the first-order dimension (CR = 5.061 > 3), it is retained despite not meeting the criteria in certain aspects. For details, please see Tables 1, 2.

**Table 1:** Item analysis of the Chinese version of the SII (first-order dimensions).

Scientific sensitivity			Scientific creativity			Scientific productivity		
Item	CR	*t*	item	CR	*t*	Item	CR	*t*
K2	12.604	0.46^**^	K3	18.893	0.63^**^	K1	5.061	0.22^**^
K7	13.822	0.50^**^	K4	17.838	0.54^**^	K5	17.635	0.60^**^
K8	22.731	0.63^**^	K9	19.208	0.61^**^	K6	20.967	0.60^**^
K12	9.464	0.34^**^	K10	20.075	0.60^**^	K11	20.477	0.62^**^
K13	13.902	0.52^**^	K15	16.571	0.60^**^	K17	18.596	0.64^**^
K14	19.980	0.59^**^	K16	19.355	0.59^**^	K20	14.921	0.56^**^
K18	18.946	0.58^**^						
K19	19.217	0.60^**^						

*p < 0.05, **p < 0.01.

**Table 2:** Item analysis of the Chinese version of the SII (second-order dimensions).

Emotional understanding			Imaginative experience			Diversity			Originality			Creation and reproduction			Scientific sense of reality		
Item	CR	t	Item	CR	t	Item	CR	*t*	Item	CR	*t*	Item	CR	*t*	Item	CR	*t*
K7	14.54	0.59^**^	K2	16.20	0.54^**^	K3	25.60	0.77^**^	K4	26.44	0.68^**^	K5	22.55	0.66^**^	K1	24.79	0.67^**^
K12	17.26	0.51^**^	K8	27.78	0.71^**^	K9	24.57	0.76^**^	K10	21.11	0.69^**^	K11	28.23	0.73^**^	K6	40.30	0.78^**^
K13	14.74	0.60^**^	K14	21.85	0.66^**^	K15	19.01	0.72^**^	K16	22.32	0.65^**^	K17	24.90	0.73^**^			
K18	25.38	0.63^**^	K19	28.08	0.68^**^							K20	18.68	0.67^**^			

*p < 0.05, **p < 0.01.

### 3.3 Exploratory factor analysis (EFA)

Exploratory factor analysis was performed on Sample 1 (for details on sample division, see Section 2.1). The KMO value was 0.857, and Bartlett’s test of sphericity yielded a chi-squared value of 1172.587 (*p* < 0.001). Therefore, the data could be used for factor analysis. Considering the original scale’s three-dimensional, six-factor structure, the principal component method was used with the number of factors constrained, and the extracted factors were rotated using the maximum variance method. The results showed that the eigenvalues of the five factors were greater than 1, and the eigenvalue of the sixth factor was 0.982, which was slightly below 1. Nevertheless, considering the cumulative variance contribution rate, the scree plot, and other indicators, as well as the theoretical importance of the factor and its eigenvalue being close to 1, this study decided to retain the factor for subsequent analysis (Field, 2024). The cumulative variance contribution rate of the six factors was 55.90%, and the factor loadings were between 0.327 and 0.824. However, the results also showed that the factors to which the items belonged had changed significantly and were inconsistent with the original scale. Therefore, after reexamining the theoretical basis of the scale and ensuring that the factor structure was consistent with the theoretical hypothesis, data were collected again, and confirmatory factor analysis (CFA) was used to test the validity of the factor structure.

It is important to note that the observed deviations in item–factor loadings do not inherently invalidate the original theoretical model. This study was designed as a theory-driven, confirmatory investigation aimed at testing the cross-cultural applicability of the established hierarchical model (three dimensions comprising six factors) proposed by Mun et al. (2015), rather than generating a new, data-driven structure. Exploratory factor analysis, while useful for initial data inspection, is limited in its capacity to formally test such complex, correlated, and hierarchical factor structures. Therefore, to appropriately evaluate the hypothesized model, we collected an independent sample (Sample 2) and proceeded with confirmatory factor analysis (CFA), which is the methodologically prescribed approach for testing *a priori* theoretical models.

### 3.4 Confirmatory factor analysis (CFA)

Confirmatory factor analysis was conducted on Sample 2 data using Mplus 8.3 (for details on sample division, see Section 2.1) with the maximum likelihood (ML) estimation method. The model fit indices were as follows (shown in Table 3): χ^2^/df = 1.553, RMSEA = 0.049, SRMR = 0.041, and CFI = 0.906. The CFI value reached the widely accepted threshold of 0.90, indicating an acceptable model fit (Hu and Bentler, 1999; Wen et al., 2004). Although this value is at the lower bound of the threshold, both RMSEA (<0.05) and SRMR (<0.08) performed excellently, well surpassing their respective stringent criteria. Recent methodological studies indicate that RMSEA is a robust indicator of absolute model fit and may, in some contexts, be more informative than CFI (Chen, 2007). Moreover, the application of fit index cutoffs should be considered flexibly (Marsh et al., 2004). Considering multiple fit indices collectively, the overall fit of the model is statistically acceptable, indicating that the revised scale demonstrates acceptable construct validity and can be used as a reference in subsequent studies.

**Table 3:** Model fit indices of the confirmatory factor analysis for the Chinese version of the SII.

Fit category	Index name	Criterion for good fit	Result	Model fit evaluation
Absolute fit indices	RMSEA	<0.08 (acceptable) <0.05 (excellent)	0.049	Excellent
	SRMR	<0.08	0.056	Acceptable
Incremental fit indices	CFI	>0.9	0.906	Acceptable
	GFI	>0.9	0.932	Acceptable
	IFI	>0.9	0.909	Acceptable
Parsimonious fit in-dices	χ^2^/df	1–3 (excellent) <5 (acceptable)	1.553	Excellent

### 3.5 Criterion-related validity

The criterion-related validity of the Chinese version of the SII was examined. The results are shown in Tables 4, 5. The Science Imagination Inventory (SII) was significantly correlated with the Vividness of Visual Imagery Questionnaire (VVIQ) (r = 0.269, *p* < 0.01). The first-order dimensions of the SII, such as scientific sensitivity, scientific creativity, and scientific productivity, were significantly correlated with visual imagination (r = 0.140 ~ 0.302, *p* < 0.01). Among the second-order dimensions of the SII, emotional understanding, imagination experience, diversity, and creative reproduction were significantly correlated with visual imagination (r = 0.212 ~ 0.304, *p* < 0.01). Originality and scientific realism were not significantly correlated with visual imagination.

**Table 4:** Examination of gender-based measurement invariance for the SII.

	Model fit						Model comparison				
Model	χ2	df	RMSEA	CFI	TLI	SRMR	Δχ2	Δdf	*p*	ΔCFI	ΔTLI
Configural invariance	543.622	326	0.064	0.885	0.866	0.063					
Metric invariance	556.946	343	0.062	0.887	0.875	0.069	13.324	17	0.95	0.002	0.009
Scalar invariance	625.786	360	0.067	0.859	0.852	0.075	68.84	17	<0.001	−0.028	−0.023

χ²: Chi-square.df: Degrees of Freedom.RMSEA: Root Mean Square Error of Approximation.CFI: Comparative Fit Index.TLI: Tucker-Lewis Index.SRMR: Standardized Root Mean Square Residual.Δχ²: Change in Chi-square (Δχ²).Δdf: Change in Degrees of Freedom (Δdf).p: p-value for the Δχ² test.ΔCFI: Change in Comparative Fit Index (ΔCFI).ΔTLI: Change in Tucker-Lewis Index (ΔTLI).

**Table 5:** Examination of grade-level measurement invariance for the SII.

	Model fit						Model comparison				
Model	χ2	df	RMSEA	CFI	TLI	SRMR	Δχ2	Δdf	*p*	ΔCFI	ΔTLI
Configural invariance	1194.754	812	0.053	0.847	0.821	0.068					
Metric invariance	1279.720	880	0.052	0.840	0.827	0.008	84.966	68	0.075	−0.007	0.006
Scalar invariance	1532.500	948	0.061	0.766	0.766	0.088	252.78	68	<0.001	−0.074	−0.061

χ²: Chi-square.df: Degrees of Freedom.RMSEA: Root Mean Square Error of Approximation.CFI: Comparative Fit Index.TLI: Tucker-Lewis Index.SRMR: Standardized Root Mean Square Residual.Δχ²: Change in Chi-square (Δχ²).Δdf: Change in Degrees of Freedom (Δdf).p: p-value for the Δχ² test.ΔCFI: Change in Comparative Fit Index (ΔCFI).ΔTLI: Change in Tucker-Lewis Index (ΔTLI).

### 3.6 Assessment of scale reliability

An internal consistency reliability test was performed on the Science Imagination Inventory utilizing Sample 3 (for details on sample division, see Section 2.1). The findings indicated that the Cronbach’s *α* coefficient for the SII was 0.860. The reliability scores for the sub-dimensions of scientific sensitivity, scientific creativity, and scientific productivity were 0.718, 0.731, and 0.621, respectively. The split-half reliability coefficient for the Science Imagination Inventory was 0.820. These findings indicate that the Science Imagination Inventory possesses an acceptable level of reliability.

### 3.7 Equivalence validation

This study examined the cross-gender equivalence of the scale using multi-group confirmatory factor analysis. As shown in Table 4, both configural invariance (χ^2^/df = 1.67, CFI = 0.885) and weak invariance (ΔCFI = +0.002) were supported, suggesting consistency in the factor structure and factor loadings across gender. However, scalar invariance was not supported, as the model fit significantly deteriorated when item intercepts were constrained to be equal.

This study examined the measurement equivalence of the scale across different grade levels using multi-group confirmatory factor analysis. As shown in Table 5, both configural invariance (χ^2^/df = 1.47, CFI = 0.847) and metric invariance (ΔCFI = -0.007, *p* = 0.075) were supported, indicating that the scale exhibited the same factor structure and factor loadings across students from different grades. However, the model did not meet the criteria for scalar invariance.

The lack of scalar invariance in both analyses indicates that the item intercepts of the scale were not equivalent across the gender and grade groups. Therefore, although the scale demonstrated consistency in the meaning of the latent construct (factor structure and loadings) across these groups, direct statistical comparisons of the observed mean scores between male and female students, or between students from different grades, are not justified and should be avoided when interpreting the results.

## 4 Discussion

### 4.1 The relevance and key characteristics of the SII-CV

The Chinese version of the Science Imagination Inventory (SII) offers three primary advantages over traditional assessment tools for evaluating scientific imagination in primary school students. First, it exhibits scientific rigor and targeted dimensionality. Traditional assessments, including the Williams Creativity Test, which evaluates general creativity, and the Mental Rotations Test, which assesses spatial cognition, seldom consider factors such as motivation, emotion, and other affective influences (Cho, 2017; Zeng et al., 2011). Grounded in the philosophy of science and cognitive psychology, the SII precisely delineates three core characteristics of scientific imagination: affective drive, creativity, and realistic possibility (French, 2020; Ganiev and Tashev, 2021; Mun et al., 2015; Oh, 2022; Savojardo, 2024). Second, the instrument demonstrates strong age appropriateness in its design. The SII is designed for children aged 7–11 years (Grades 2–6), employing straightforward language in its items while avoiding complex terminology. This approach intentionally omits task types, such as those used in mental rotation tests, which depend significantly on abstract spatial reasoning. This alignment with the cognitive development patterns of this age group addresses the limitations of traditional assessment tools for younger populations. Third, the SII provides detailed assessment results. The scale includes three primary dimensions: scientific sensitivity, scientific creativity, and scientific productivity, along with six secondary factors. It provides an overall assessment of scientific imagination while enabling the identification of specific areas of weakness, such as limited realism in scientific reasoning or diminished originality, through its sub-dimensional profiles. This level of precision provides clear guidance for future interventions, addressing the shortcomings of conventional tools, which typically yield only overall scores without detailed diagnostic information (Murphy, 2020; Zabelina and Condon, 2020).

The Chinese version of the SII effectively achieves the intended goals of “scientific measurement + practical feedback” from the evaluation effect perspective. The assessment results demonstrated strong reliability and validity. Item analysis indicated that all items effectively discriminated between the high and low groups, with significant differences observed (*p* < 0.01), thereby accurately identifying varying levels of scientific imagination and addressing the issue of score interpretation. In addition, criterion validity analysis revealed significant positive correlations between the scale dimensions and the VVIQ (r = 0.14–0.30, *p* < 0.01), indicating that the assessment results closely align with students’ imaginative performance and accurately reflect their scientific imagination levels. Conversely, the practical implications of the feedback results indicate that the scale can not only provide “high and low scores” but also interpret the underlying reasons for these scores through score analysis (for instance, a low sense of scientific realism may stem from cognitive development or cultural influences). Teachers can use the interpretations to design targeted intervention strategies, while researchers can propose targeted training recommendations based on the findings. This approach transforms assessment from a mere measurement into a practical tool that fosters the development of scientific imagination, thereby achieving the outcome of assessment as guidance.

### 4.2 Implications of scalar non-invariance: defining the scope of valid use for SII-CV in China

The Chinese version of the SII demonstrated satisfactory reliability and validity, confirming its fundamental utility for assessing scientific imagination within the sampled Chinese elementary school context. A pivotal finding of this rigorous validation process, however, was the failure to achieve scalar invariance across gender and grade levels. This result is not merely a limitation but also a crucial empirical finding that informs the appropriate and nuanced application of the scale in China.

The lack of scalar invariance indicates that students of different genders and grade levels may respond systematically differently to certain SII items, despite possessing equivalent levels of the underlying trait (e.g., scientific sensitivity). This pattern of response bias likely reflects underlying cultural and socialization influences. In particular, the absence of scalar invariance across gender (ΔCFI = −0.028) suggests that gender role expectations in Chinese society may influence how students respond to certain items (Wei et al., 2024). For example, in the “scientific sensitivity” dimension, girls may systematically provide higher scores on items related to emotional understanding and empathy, even when boys and girls have the same level of potential traits. This pattern likely reflects the influence of socialization, where girls’ higher scores are not necessarily indicative of higher latent trait levels, but may instead reflect a greater social comfort with acknowledging emotions—a skill cultivated through gendered socialization practices (Eagly and Wood, 2013; Gui, 2019; Portela-Pino et al., 2021; Wei et al., 2024). Conversely, prevailing stereotypes in science education that associate objectivity and rationality with masculinity may discourage boys from engaging with or reporting the emotional aspects of scientific inquiry, leading to an underestimation of their abilities on such items and posing a threat to the validity of mean comparisons (Gong et al., 2018; Skipper and Fox, 2022).

The lack of scalar invariance across grade levels (ΔCFI = −0.074) suggests that younger and older students may interpret or respond to certain items differently, even when they possess equivalent levels of the underlying scientific imagination trait. This measurement bias can likely be attributed to a combination of developmental and educational factors. A clear example is observed in the ‘scientific productivity’ dimension, where second-grade students consistently rated items related to ‘scientific sense of reality’ significantly lower compared to their third- to sixth-grade counterparts. This pattern may be explained by several interrelated factors: (1) cognitive development. Younger students (e.g., second graders) operate primarily at a concrete operational stage, which makes it challenging for them to evaluate the “scientific sense of reality” in hypothetical scientific scenarios—tasks that require more abstract and hypothetical thinking, abilities that develop with age (Denton et al., 2022). (2) Exposure to the formal science curriculum. Younger students have had less exposure to the formal science curriculum that provides the knowledge base for such judgments (Curran and Kitchin, 2019; Kaderavek et al., 2020). (3) Linguistic and reading comprehension ability. Items designed to assess “creation and reproduction” may contain vocabulary or syntactic structures that impose a higher cognitive load on younger children with less developed reading skills (Denton et al., 2022; Smith et al., 2021). This could cause younger students to systematically underestimate an item’s feasibility due to incomplete comprehension, rather than reflecting a true lack of scientific imagination.

Therefore, the primary contribution of this validation study extends beyond confirming the basic factor structure. It empirically defines the boundaries of valid score interpretation: The SII-CV is robust for assessing individual differences, tracking development over time within the same demographic group, and examining correlations with other variables. However, the observed measurement non-invariance clearly indicates that it is not psychometrically appropriate for direct mean comparisons across gender or grade groups. This precise delineation prevents the misuse of the scale and ensures that future research using the SII-CV produces conclusions that are both methodologically sound and culturally informed.

### 4.3 Limitations

The present study has several limitations that should be considered when interpreting its findings and planning future research. First, the use of a convenience sample from a limited number of primary schools in Suzhou constrains both the diversity of the sample and the generalizability of the results. Although efforts were made to include schools from different districts and of different types, the findings primarily reflect an urban context in eastern China and may not extend to rural populations or other regional settings. Second, the psychometric evaluation revealed that certain items, although theoretically meaningful, contributed to suboptimal reliability indices. These items were retained to preserve content validity and theoretical integrity, but their impact on measurement precision warrants further investigation. The most notable methodological limitation pertains to measurement invariance: while configural and metric invariance were established across gender and grade groups, scalar invariance was not achieved. This indicates that, although the factor structure and factor loadings are comparable across groups, differences in item intercepts prevent statistically justified direct comparisons of the observed mean scores between these demographic categories. Consequently, the scale in its current form is not suitable for group-difference studies. Future research should recruit more representative and geographically diverse samples across China and investigate the causes of scalar non-invariance, such as cultural response patterns or developmental differences in item interpretation.

## 5 Conclusion

This study shows the reliability and validity of the Chinese version of the SII for evaluating scientific imagination in primary school students. It provides a timely, developmentally appropriate measurement of scientific imagination among primary school students in mainland China. However, the absence of full scalar invariance necessitates careful consideration and the use of clear guidelines when applying the scale in group-based research.

## Data Availability

The raw data supporting the conclusions of this article will be made available by the authors, without undue reservation.
